# Effective Concentration and Detection of *Cryptosporidium*, *Giardia*, and the Microsporidia from Environmental Matrices

**DOI:** 10.1155/2014/408204

**Published:** 2014-09-10

**Authors:** Joseph A. Moss, John Gordy, Richard A. Snyder

**Affiliations:** ^1^Center for Environmental Diagnostics and Bioremediation, University of West Florida, 11,000 University Parkway, Building 58, Room 70, Pensacola, FL 32514, USA; ^2^Water Quality Laboratory, City of Tampa Water Department, 7125 North 30th Street, Tampa, FL 33610, USA

## Abstract

*Cryptosporidium* spp., *Giardia* spp., and members of Microsporidia are enteropathogenic parasites of humans and animals, producing asymptomatic to severe intestinal infections. To circumvent various impediments associated with current detection methods, we tested a method providing multistage purification and separation in a single, confined step. Standard real-time PCR was used as a detection method. Samples spiked with *C. parvum* and *G. intestinalis* were split for comparison to standard Method 1623. Results were equivalent to immunomagnetic procedures for *Cryptosporidium*, and *Giardia*. Overall percent recovery for *Cryptosporidium* with Method 1623 averaged 26.89% (std 21.44%; min = 0%; max = 73%) and was similar but less variable for qPCR method at an estimated average of 27.67 (std 17.65%; min = 5%; max = 63%). For *Giardia*, Method 1623 had an overall average recovery of 27.11% (std 17.98%; min = 1%; max = 58%), while multistage purification and qPCR had an estimated lower overall recovery at 18.58% (std 13.95%; min = 0%; max = 35%). Microsporidia were also readily detected with an estimated recovery of 46.81% overall (std 17.66%; min = 18%; max = 70%) for *E. intestinalis* and 38.90% (std 14.36%; min = 13%; max = 62%) for *E. bieneusi*.

## 1. Introduction

*Cryptosporidium, Giardia*, and Microsporidia are enteropathogenic parasites of humans and animals, producing asymptomatic to severe intestinal infections [1, 2]. Detection of these pathogens continues to be of great interest for public health, and direct detection monitoring is warranted given a poor correlation with standard fecal pollution indicators [3]. Currently, the U.S. and several European nations mandate the use of combined immunomagnetic and microscopy-based (IMS) procedures (i.e., IT rule; Method 1623) for monitoring surface and drinking waters for* Cryptosporidium* and* Giardia*. These methods are hampered by cost and their time consuming nature [4] and require specific expertise to distinguish between human and animal pathogenic* Cryptosporidium* and* Giardia* species [5]. Problems also arise with false positive and negative results [6] and poor dissociation of oocysts from magnetic beads in the purification step [7]. These methods are specific only to* Cryptosporidium* and* Giardia*, and though Microsporidia and other waterborne pathogens are listed in various contaminate lists, no cost and time-effective method for their detection exists.

It is well known that molecular techniques have been developed that are more effective than immunofluorescence microscopy in detecting specific pathogens [8]. Notably, recreational and environmental waters, such as surface waters and areas impacted by sewage outfalls present complex sample matrices known to contain numerous organic and inorganic dissolved and particulate substances that can affect sample collection and purification as well as having the potential to inhibit PCR reactions [6]. Many current molecular-based methods rely upon IMS to alleviate these problems. The IMS technique is not without difficulties; highly turbid samples affect pathogen recovery [9] resulting in either low and/or variable rates [7]. In addition, nonspecific binding of antibodies in complex samples appears to be a factor limiting detection sensitivities. Therefore, affordable alternative methods are needed to help bolster pathogen detection in water matrices.

To circumvent problems associated with IMS, a basic method combining filter dissolution and separation of targets in a centrifugation device (CFD) was evaluated. The procedure was coupled with downstream qPCR for a rapid, cost-effective approach to detecting these pathogens. This report describes the procedure and presents data regarding sensitivity and efficacy in a comparative evaluation to use of IMS microscopy performed in a NELAC certified laboratory.

## 2. Materials and Methods

### 2.1. Pathogens

Inactivated* Cryptosporidium parvum* oocysts (CpAZ strain) and* G. intestinalis* (H3 isolate, assemblage B) cysts (gamma-irradiated) used for spiking suspension in all trials (Method 1623 and qPCR) and standards (qPCR). Pathogens were flow cytometry-sorted and suspended in 0.75 mL of reagent water with 0.01% Tween 20, which had been inactivated and preserved (Accuspike-IR; Waterborne Inc., LA).* Encephalitozoon intestinalis* were supplied as purified suspensions in phosphate-buffered (PBS) solution (1 × 10^6^ spores; Didier isolate; P103I) and* Enterocytozoon bieneusi* spores were supplied as unpurified suspensions (Waterborne Inc; New Orleans, LA). All oocysts, spores, and cysts were purchased 3-4 weeks prior to trials and stored at 4°C. All parasites are evaluated for general quality and intactness under differential interference contrast microscopy.

### 2.2. Comparative Trials

For a comparative evaluation with IMS-microscopy (Method 1623; [10]), bulk volumes of water of varying turbidity (<1 to 54 NTUs) were divided into separate 20–25 L volumes and spiked with known quantities of* C. parvum* oocysts and* G. intestinalis* cysts (Accu-spike; Waterborne Inc. New Orleans, LA). To standardize turbidity treatments and to ensure the absence of pathogens in water samples prior to spiking, water sample volumes having turbidities > 1 were created by adding a purified loam of soil to Milli-Q purified water (18 MΩ; Millipore, Bedford, MA). Soil was autoclaved twice at 121°C for 20 min at 24 hr intervals prior to mixing with water. Water used for spiking with turbidity standards < 1 NTUs (potable water) was provided at the City of Tampa Water Department (Biol. Dept).

Water of varying turbidities was divided into separate polycarbonate carboys (20–25 L). In each of 2 trials, sample water of 3 turbidities (3 replicates/turbidity) was spiked with 100 (oo)cysts each of* C. parvum* and* G. intestinalis.* Prior to spiking, representative aliquots from carboys were tested for turbidity using a turbidimeter (2100N, Hach). Carboys were spiked with gamma-irradiated* C. parvum* oocysts and* G. intestinalis* cysts according to the manufacturer's instructions (AccuSpike; Waterborne Inc, LA). Additionally, carboys were spiked with predetermined counts of* E. intestinalis* and* E. bieneusi* for evaluation of separation via the CFD and detection using qPCR. Estimates for spiking suspensions and for qPCR standard curves were done via hemocytometer counts. The standard suspensions of Microsporidia were prepared 1 week prior to each trial. Method blanks, in which no pathogens were added, were included in trial 1.

Spiked samples evaluated using Method 1623 were filtered through FiltaMax filters (Idexx) with flow volumes recorded using a digital rate meter (model 220/101-8T Flo-Sensor; McMillan). Sample volumes evaluated using filter dissolution combined with qPCR were collected on 142 mm mixed cellulose ester (MCE; 1.2 um porosity; Millipore) membranes using a filter tower (5 mm Hg vacuum). MCE filters were folded, placed into zip-lock bags, and labeled accordingly. Filters were transported to UWF on ice and processed within 48 hrs.

### 2.3. Environmental Samples

Environmental samples were collected from the Pensacola and the Tampa Bay region. A few additional samples were collected from rivers and waste water treatment facilities (WWTF) in St. Petersburg and Auburndale, Florida. Samples from the Pensacola region were collected into polycarbonate carboys and transported back to the lab for filtering and processing. Unused portions of samples processed by routine testing at the City of Tampa Water facility by Method 1623 were used for evaluation via qPCR. Unused volumes were filtered onto 1.2 *μ*m MCE filters which were shipped overnight to UWF. The volumes were not equal but provided additional testing material.

### 2.4. Method 1623

Samples were processed as described for the IMS microscopy method (Method 1623; [10]) at the Water Quality Laboratory at the City of Tampa Water Department. The Dynal IMS procedure (Dynabeads G/C combo IMS kit; Dynal A.S., Oslo, Norway) was performed according to the manufacturer's instruction. Adhesion slide preparations were immunostained with fluorescein isothiocyanate (FITC) labeled antibody (Meridian Biosciences) following manufacturer's instructions and stained with 4′,6-diamidino-2-phenylindole (DAPI) 0.001 mg/mL (Sigma-Aldrich) according to Method 1623. Enumeration of oocysts and cysts was accomplished using an Olympus BX60 microscope with magnifications of 200x–1000x for the examination of immunofluorescence (FA), DAPI staining characteristics, and differential interference contrast (DIC) microscopy according to Method 1623.

### 2.5. Centrifugation-Filtration Purification

**Figure 1:**
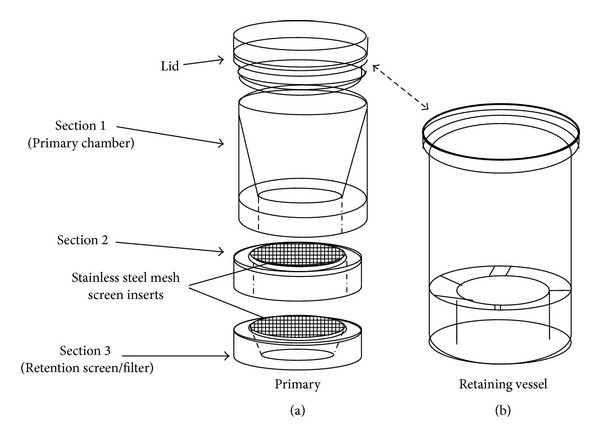
Illustration of centrifugation-filtration device (CFD) utilized.

The study utilized a multitiered centrifugation-filtration (CFD; GenIUL Terrassa, Spain) device (Figure 1) which consists of interlocking (vapor-tight) sections for separation of large particulates and for collection of target organisms. Prior to filter dissolution, a 47 mm solvent resistant “retention” filter (2 *μ*m; Nuclepore Track-Etched, Whatman) was placed upon the mesh screen of the lower section (Section  3, Figure 1(a)) to collect target pathogens; 47 mm prescreens (20 *μ*m; Nytex) were placed upon mesh supports to remove larger particulates.

For dissolution, the MCE filters were folded using sterile forceps and inserted into the primary chamber (Section  1; Figure 1(a)) of the CFD. This was followed by addition of ~30 mL acetone (95–100%) and ~25 zirconia/silica beads (2.3 mm; BioSpec Inc.). The lid was secured on the primary apparatus which was then fastened into its retaining vessel. The unit was thoroughly agitated (hand shaking; ~1 min) and centrifuged at ~2000–3600 rcf for 3–5 min (Sorvall RC-5B/C with a GSA rotor; DuPont). The primary unit was then removed from its retaining vessel and flow-through discarded. Two additional wash steps using acetone (100%) and centrifugation (2000–3600 rcf for 2-3 min) were performed. Following completion of wash steps, the primary unit was inspected to ensure that no residual dissolution fluid remained on the retention filter. When necessary, residual fluid was removed via additional centrifugation and/or by application of vacuum to the bottom of the unit by a filtration manifold.

### 2.6. gDNA Extraction

Retention filters were removed from CFD units with sterile forceps and inserted into separate 2 mL, bead-beading tubes (Powersoil; MoBio). Lysis buffer was added and filters were subjected to 3 freeze-thaw (liquid nitrogen/65°C) cycles. Tubes were agitated using a FastPrep (ThermoSavant) or a PowerLyzer homogenizer (MoBio) using 45 second bursts at setting 4.5 and S3500, respectively. Genomic DNA extraction was done according to the manufacturer's instructions and stored at −20°C.

### 2.7. Amplification and Detection

Primer-probe sets which were used to detect* G. intestinalis* cysts and* C. parvum* oocysts consisted of those targeting the *β*-Giardin gene and the gene for the* Cryptosporidium* oocyst outer wall protein (COWP; [11]). PCRs were performed in 15 *μ*L volumes containing 3.0 mM MgCl_2_, PCR buffer (FastTaq 10x/Green), 0.50 *μ*M each primer, 0.04 U of FastStart* Taq* DNA polymerase (Roche), 0.2 mM PCR Nucleotide MixPlus (Roche), 0.025% bovine serum albumin (Sigma, St. Louis, Mo.), and 0.3 *μ*M of each hybridization probe. Cycling conditions were consistent with those of Guy et al. 2003. Fluorescence was measured at the end of each cycle. For detection of* E. bieneusi* spores, primers, and hybridization probe targeting the ITS region of the 18S rRNA gene were utilized [12]. Primers and probe used for detection of* Encephalitozoon* spp. (pan-*Encephalitozoon*) spores were from Wolk et al. 2002 [13]. Reactions for detection of* E. bieneusi* and* Encephalitozoon* spp. were performed separately in 15 *μ*L total volumes containing 3.0 mM MgCl_2_ (Roche), PCR buffer (FastTaq 10x/Green), 0.40 *μ*M each primer, 0.04 U of FastStart* Taq* DNA polymerase (Roche)/l, 0.2 mM PCR Nucleotide MixPlus (Roche), 0.025% bovine serum albumin (Sigma, St. Louis, Mo.), and 0.3 *μ*M of each hybridization probe. Thermal conditions for both reactions consisted of 5 min at 95°C followed by 50 cycles of 15 s at 95°C, 30 s at 60°C, and 30 s at 72°C. Fluorescence was measured at annealing step of each cycle.

A minimum of 8–10 replicate reactions were used for all samples and results pooled. To ensure purity of reagents, “no template controls” (NTCs) were run with every PCR reaction series. Standard suspensions containing 100 and 500 cysts and oocysts and 1000 and 5000 spores were used to develop standard curves. Standards were subjected to gDNA extraction methodology as stated previously. The projected number of pathogens was calculated based on the portion of gDNA analyzed using the following formula: number of pathogens (i.e., oocysts, spores) in each sample = (number of pathogens calculated) × (total gDNA extraction volume)/(total volume of gDNA analyzed). All runs and data analysis were performed using Rotor-Gene 3000 (Corbett) real-time PCR machines and software. Pathogen presence and species identification in unknowns were verified using electrophoresis on 2% agarose gels and subsequent sequencing (Big Dye; V1.1; ABI 3100 sequencer) according to manufacturer's instructions. Data were aligned against existing sequences for known pathogens (BLAST; NCBI).

## 3. Results

**Figure 2:**
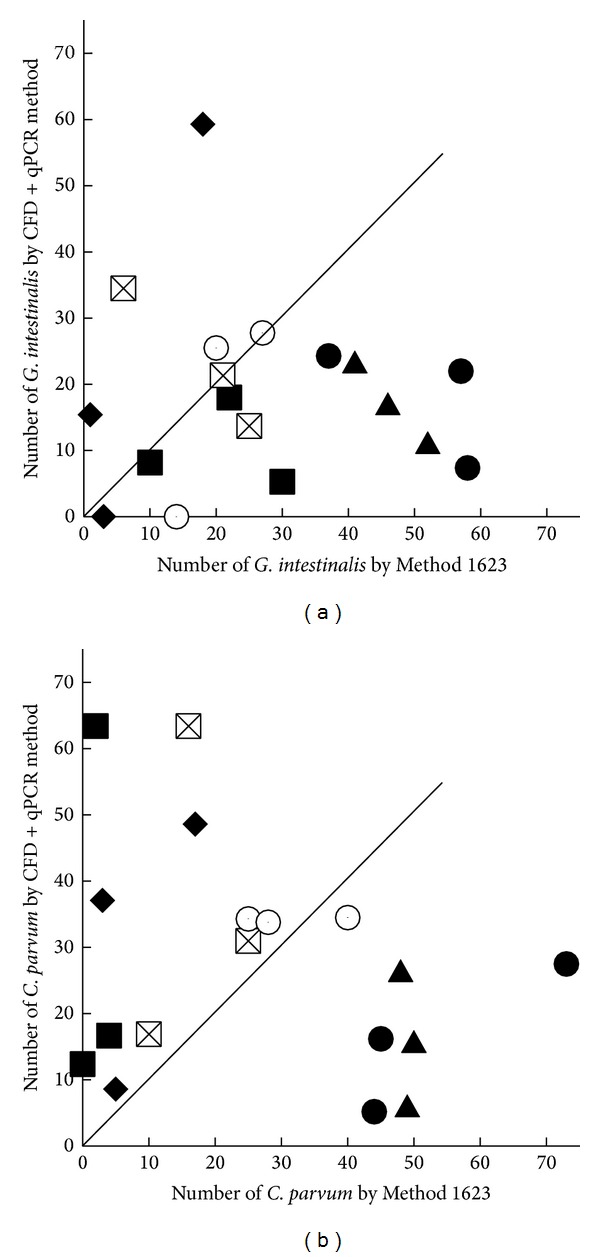
A comparison of split sample analysis at various turbidity amounts by Method 1623 and the CFD + qPCR method for* Giardia intestinalis* (a) and* Cryptosporidium parvum* (b). The solid lines represent a 1 : 1 correspondence of results. Solid circles: tap water 0.2 NTU, open circles: tap water 0.4 NTUs, x-squares: 1.1 to 1.3 NTUs, triangles: 3.8 NTUs, squares: 7.1 NTUs, and diamonds: 54 NTUs.

Using inactivated (gamma-irradiated)* C. parvum* oocysts and* G. intestinalis* cysts, parallel processing of split samples directly compared Method 1623 and qPCR. Data for the methods comparison are listed and presented (Table 1; Figure 2). Overall percent recovery for* Cryptosporidium* with Method 1623 averaged 26.89% (std 21.44%; min = 0%; max = 73%) and was similar but less variable for the CFD + qPCR method at an average of 27.67 (std 17.65%; min = 5%; max = 63%). For* Giardia*, Method 1623 had an overall average recovery of 27.11% (std 17.98%; min = 1%; max = 58%), while the CFD + qPCR had a lower overall recovery at 18.58% (std 13.95%; min = 0%; max = 35%).

Microsporidian spores,* E. intestinalis,* and* E*.* bieneusi* spores were added to samples as well as* C. parvum* and* G. intestinalis*. The estimated number of* E. intestinalis* and* E. bieneusi* spores detected using CFD + qPCR and associated percent recovery for each sample are listed (Table 1). The percent recovery for the Microsporidia by qPCR was 46.81% overall (std 17.66%; min = 18%; max = 70%) for* E. intestinalis* and 38.90% (std 14.36%; min = 13%; max = 62%) for* E. bieneusi* (Table 1). Use of two 142 mm MCE filters was necessary for samples having a turbidity of 54 NTUs. Additional agitation time (~2 min) was necessary to allow for complete dissolution of the filters as well as supplementary rinse steps (2-3 times) for removing additional cellulose.

**Figure 3:**
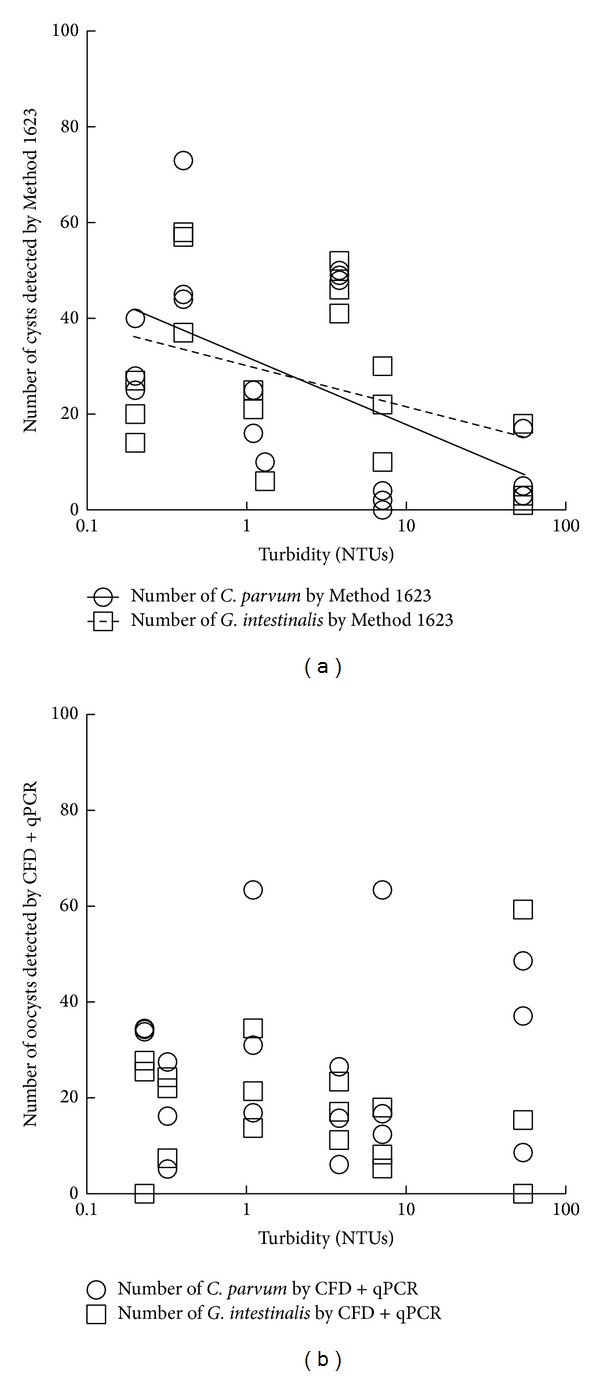
The effect of increasing turbidity in samples on the performance of Method 1623 (a) and the CFD + qPCR method (b) for both* Giardia intestinalis* and* Cryptosporidium parvum. *

**Figure 4:**
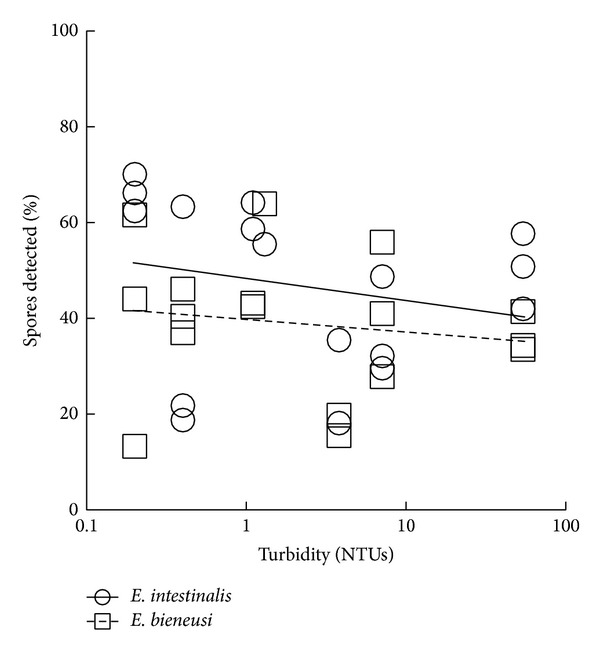
Detection efficiency of* E. intestinalis* and* E. bieneusi* by CFD + qPCR with increasing turbidity.

A decline in the ability of Method 1623 to detect* Cryptosporidium* and* Giardia* cysts was observed with increasing turbidity, while the qPCR method was less affected by turbidity for detection of* Cryptosporidium* and* Giardia* (Figure 3) and* E. intestinalis* and* E. bieneusi* (Figure 4).

Data from qPCR using samples volumes left over after employment of Method 1623 is presented in Table 2. Use of qPCR enabled detection of* Giardia intestinalis* and Microsporidian pathogens in 80% of the environmental samples evaluated.* Cryptosporidium* numbers fell below detection thresholds. The majority of pathogens detected were primarily Microsporidia, with* E. hellem* in the majority of environmental and waste water samples.

## 4. Discussion

Finding the origin of fecal pollution is paramount in assessing associated health risks. However, microbial source tracking (MST) is often expensive, time-consuming, and intimidating to those who need it [14]. Most approaches are not logistically feasible to real-world situations and difficult to gauge for their effectiveness due to the fact there is no regulatory method for making comparisons [15]. This is particularly the case for PCR-based methodologies designed for detection, verification, and relative quantification of pathogens in environmental samples. MST methods need to be chosen using consideration of cost, reproducibility, discriminatory power, ease of interpretation, and ease of performance [16, 17]. Limitations of microscopy, including slow analysis time and inability to differentiate pathogen species and strains, have been rectified by molecular-based methodologies which have also shown higher sensitivity than Method 1623 and the United Kingdom regulatory method [18, 19].

In the event of large outbreaks, analysis of numerous field samples to provide spatial and temporal assessments is necessary [20]. Arguably, the high cost (>$400/sample; EPA-certified labs) associated with IMS-microscopy based detection prohibits such use and constrains from implementing routine monitoring. Costs incurred in this study were approximately $40/sample and include costs for filters, gDNA extraction, and qPCR reagents and hybridization probes. The CFD methodology worked at a fraction of the cost (12%) and time (6 times faster), based on evaluation of membrane filtration and IMS methodologies ([21]; this study). The methodology was shown to be effective in detection of pathogens present at moderate concentrations (≥5 (oo)cysts/L) or higher. Isolation of pathogens present in the environments at lower concentrations may still prove to be inconstant, though the specificity of qPCR is high with low time consumption in contrast to visual methods. Additionally, the methodology used herein may aid in detection of various pathogens as commercial IMS kits do not permit collective isolation of multiple pathogens from single samples and have not been developed for other known pathogens (i.e.,* Cyclospora cayetanensis*). Regardless, a combination of molecular and microscopy-based analyses should ultimately increase effectiveness of waterborne pathogen detection.

The purification approach in this study is an adaptation of the cellulose-acetate filter dissolution, having previous reported rates of recovery ranging from 70–79% for the recovery of* Cryptosporidium* spp. oocysts [22, 23]. Mean recoveries of 50.2% for* Cryptosporidium* oocysts and of 63.1% for* Giardia* cysts have also been reported [24]. The methodology has been used with microscopy [25] as well as PCR [26] and fluorescent* in situ* hybridization (FISH) [27]. However, losses have been attributed to the multiple centrifuge and aspiration steps, as well as the occurrence of a hardened pellet which made the process of detection of pathogens more difficult. These steps have been circumvented in this study, reducing the process to one combined step and bypassing the need for aspirating and pelleting of sample material and associated solvent vapor exposure.

Concerns over providing information on the viability and infectivity of protozoan pathogens have been made [24, 28]. However,* C. parvum* oocysts were reported to be viable in mice following use of the methodology [29]. These issues may be addressed through use of propidium monoazide prior to acetone dissolution and PCR [30]. In any case, continual detection of a high concentration of pathogens in a sample despite capacity to confirm pathogenicity or viability should be of concern.

The multiplex primer-probe set [11] enabled evaluation of the CFD methodology and allowed for simultaneous amplification of both targets within a single reaction tube. Detection of ~5 (oo)cysts/L in water samples having turbidities of 3.8–54 NTUs was achievable; providing detection sensitivities which fall into acceptable limits with respect to reported ID-50s of* G*.* intestinalis* (25–100 cysts; [31, 32]) and* C*.* parvum* (10–132 oocysts, [33–35]) and estimates of average consumption of recreational water while bathing (~79.7 mL, [36]; ~50 mL, [37]).

The methodology was successful in detection of pathogens in the spiked samples as well as in environmental samples.* C. parvum* was detected in a sample of raw sewage using qPCR, and other samples for sewage and sewage treatment plant effluents were positive for Microsporidia by qPCR.* E. bieneusi* was detected in a few of the samples collected from various freshwater sources. This pathogen is prevalent throughout the world and is found in a wide variety of hosts including pigs, humans, and other mammals [38, 39]. Their presence, associated with waterborne outbreaks and also with recreational and river water, is continually being documented [36].

The number of samples and analytical replicates affects detection of pathogens in water by both microscopy and PCR [20]. A minimum of 10 replicate qPCR reactions were pooled to improve analytical sensitivity by accounting for the probability of missing target sequences in the aliquots taken from gDNA extract solutions [40]. Increasing the number of PCR reactions increases sensitivity. Various additional primer-probe combinations directed at detection of* Cryptosporidium* [41] and* Giardia* [42] currently exist, and certain primer-probe combinations may prove to work better. More importantly, recent advances like digital-droplet PCR should ultimately improve overall detection sensitivity. Reduction from 20 *μ*m to 12–15 *μ*m prescreens may improve the overall performance of the method. Nytex was used since, at the time, no stainless steel mesh prescreen was available. Additionally, gamma-irradiated, flow cytometry-sorted* C. parvum* and* G. intestinalis* suspensions (AccuSpike-IR, Waterborne Inc) were used in order to provide precision with regard to matrix spiking but also to provide a degree of safety during the development and evaluation of the assay. The effect of the gamma-irradiation on the performance of PCR is an unknown factor.

## 5. Conclusion

This report has demonstrated the capability of combining filter dissolution with qPCR for the direct detection of human enteric pathogens with results that are comparable to IMS microscopy, at substantial reductions in time and expense. Assay times were roughly 3–3.5 hr (1 technician/thermocycler, 1–6 samples) from initial filtration of samples to completion of qPCR. The method was straightforward and minimized sample manipulation.

## Figures and Tables

**Table 1:** Comparison of Method 1623 with CFD-PCR results of spiking trials using 100 *C. parvum *oocysts and 100 *G. intestinalis *cysts and about 1000 spores of *E. bieneusi *and *E. intestinalis*, respectively.

	Batch number	Method 1623 Sample data				*C. parvum* + *G. intestinalis *			CTD-qPCR Sample data				*C. parvum* + *G. intestinalis *			*E. intestinalis *			*E. bieneusi *		
		Replicate number	Volume (L)	Turbidity (NTUs)	Number of filters used	Number of (oo)cysts spiked^a^	Number of *C. parvum* detected^b^	Number of *G. intestinalis* detected^b^	Replicate number	Volume (L)	Turbidity (NTUs)	Number of filters used	Number of (oo)cysts spiked^a^	Number of *C. parvum* detected^c^	Number of *G. intestinalis* detected^c^	Number of *E. intestinalis* spiked^d^	Number of *E. intestinalis* detected^c^	Percent recovery^e^	Number of *E. bieneusi* spiked^d^	Number of *E. bieneusi* detected^c^	Percent recovery^e^
Trial 1	1	1	26.5	1.1	1	100	25	25	1	20	1.1	1	100	31	14	1050 ± 20	674	64	1232 ± 54	523	42
		2	22.5	1.3	1	100	10	6	2	20	1.1	1	100	17	35	1050 ± 20	583	56	1232 ± 54	788	64
		3	24.8	1.1	1	100	16	21	3	20	1.1	1	100	63	21	1050 ± 20	616	59	1232 ± 54	532	43
	2	1	21.0	7.1	1	100	4	10	1	20	7.1	1	100	17	8	1050 ± 20	512	49	1232 ± 54	505	41
		2	20.8	7.1	1	100	2	30	2	20	7.1	1	100	63	5	1050 ± 20	338	32	1232 ± 54	343	28
		3	20.3	7.1	1	100	0	22	3	20	7.1	1	100	12	18	1050 ± 20	311	30	1232 ± 54	689	56
	3	1	30.0	54.0	2	100	17	18	1	20	54.0	2	100	49	59	1050 ± 20	441	42	1232 ± 54	423	34
		2	23.0	54.0	2	100	3	1	2	20	54.0	2	100	37	15	1050 ± 20	534	51	1232 ± 54	510	41
		3	22.8	54.0	1	100	5	3	3	20	54.0	2	100	9	0	1050 ± 20	606	58	1232 ± 54	413	34
		MB	20.7	54.0	1	0	0	2	MB	20	54.0	2	0	0	0	0	0	0	0	0	0

Trial 2	1	1	21.8	0.2	1	100	25	27	1	22	0.2	1	100	34	28	1124 ± 78	788	70	935 ± 25	576	62
		2	20.3	0.2	1	100	40	20	2	22	0.2	1	100	35	26	1124 ± 78	702	62	935 ± 25	412	44
		3	22.2	0.2	1	100	28	14	3	22	0.2	1	100	34	0	1124 ± 78	744	66	935 ± 25	124	13
	2	1	23.0	0.3	1	100	73	57	1	22	0.3	1	100	28	22	1124 ± 78	712	63	935 ± 25	431	46
		2	21.3	0.3	1	100	44	58	2	22	0.3	1	100	5	7	1124 ± 78	211	19	935 ± 25	346	37
		3	20.3	0.3	1	100	45	37	3	22	0.3	1	100	16	24	1124 ± 78	245	22	935 ± 25	378	40
	3	1	21.0	3.8	1	100	49	52	1	22	3.8	1	100	6	11	1124 ± 78	ND	NA	935 ± 25	ND	NA
		2	23.0	3.8	1	100	48	46	2	22	3.8	1	100	27	17	1124 ± 78	399	35	935 ± 25	145	16
		3	21.8	3.8	1	100	50	41	3	22	3.8	1	100	16	23	1124 ± 78	204	18	935 ± 25	185	20

^a^Number of *C. parvum* and *G. intestinalis* (oo)cysts spiked using AccuSpike-IR suspensions.

^b^Number of (oo)cysts detected in entire sample.

^c^Estimated number of pathogens in sample (number of pathogens calculated × total volume of gDNA)/(volume of gDNA analyzed).

^d^Estimated number of spores spiked based on hemocytometer counts.

^e^Estimated percent recovery (number of spiked/number of detected × 100).

ND = not done; NA = not applicable; MB = method blank.

**Table 2:** Pathogen detection through Method 1623 and CFD + qPCR in environmental samples.

Environmental samples	Method 1623					CFD + qPCR			
Sample	Date	Volume (L)	*C. parvum* ^ a^	*G. intestinalis* ^ a^	(oo)Cysts per L^b^	Vol. (L)^c^	Pathogen(s) detected^d^	Number of pathogens^e^	Pathogens per L^f^
City of Auburndale WWTF	10/12/2010	50.0	8	572	0.2/11	21	*G. intestinalis* and *E. bieneusi *	122/634	6/30
Intake FL6290327 DLTWTF	10/12/2010	48.5	0	0	0/0	8.4	0	0	0/0
Lake Wales Utilities Sam P. Robinson Reclaimed WTP	11/3/2010	50.0	0	0	0/0	8	*E. intestinalis* and *E. hellem *	312	0/0
City of St. Petersburg 401 ASRWRF Inj. Well	12/13/2010	50.3	21	78	0.4/1.5	10.4	*E. bienusi *	1286	124
Intake FL6290327 DLTWTF	1/11/2011	48.8	0	2	0/0.04	5	0	0	0
Lake Wales Utilities Sam P. Robinson Reclaimed WTP	1/12/2011	40.5	89	291	2/7	4	*G. intestinalis *	105	26
City of St. Petersburg 401 ASRWRF Inj. Well	2/1/2011	50.3	24	17	0.5/0.3	19.5	*E. bieneusi *	754	39
City of St. Petersburg 764 ASRWRF B-4 Mon. Well	2/1/2011	50.4	0	0	0/0	19.3	*E. bieneusi *	538	28
City of Tampa Howard F Curren AWTP	2/2/2011	50.3	2	4	0.04/0.08	22	0	0	0
City of St. Petersburg MW401 ASRWRF B-10 Mon. Well	2/15/2011	50.7	0	0	0/0	20	*E. intestinalis* and *E. hellem *	677	34
Intake FL6290327 DLTWTF	2/2/2011	50.3	0	2	0/0.04	15.5	*E. hellem *	1515	98

^a^Number of oocysts/cysts per detected in sample.

^b^Approximate number of oocysts/cysts per liter.

^c^Sample volumes remaining after Method 1623 analysis.

^d^Pathogens detected in sample using hybridization probes and verified via downstream sequencing.

^e^Estimated number of pathogens in sample (number of pathogens calculated × total volume of gDNA)/(volume of gDNA analyzed).

^f^Approximate number of respective pathogens per liter.
